# Towards a Mathematical Structure of Global Phenomenal Consciousness

**DOI:** 10.3390/e28060615

**Published:** 2026-05-29

**Authors:** Zoe Lee-Youngzie, Naotsugu Tsuchiya, Michael Robinson, Donna Dietz, Martin M. Monti

**Affiliations:** 1 Department of Psychology, University of California, Los Angeles, CA 90095, USA; monti@ucla.edu; 2 School of Psychological Sciences, Monash University, Melbourne 3800, Australia; naotsugu.tsuchiya@monash.edu; 3 Center for Information and Neural Networks (CiNet), National Institute of Information and Communications Technology (NICT), Osaka 565-0871, Japan; 4 Laboratory of Qualia Structure, ATR Computational Neuroscience Laboratories, 2-2-2 Hikaridai, Kyoto 619-0288, Japan; 5 Department of Mathematics and Statistics, American University, Washington, DC 20016, USA; michaelr@american.edu (M.R.); dietz@american.edu (D.D.); 6 Brain Injury Research Center, Department of Neurosurgery, University of California, Los Angeles, CA 90095, USA

**Keywords:** consciousness, qualia structure, phenomenal unity, category theory, sheaf theory, mathematized phenomenology, structural approach

## Abstract

Recent work in the structural approach to consciousness has shown great promise as a research paradigm for the formal and empirical study of the phenomenal qualities of experience, i.e., *qualia*. In this paradigm, qualia are characterized by modeling the internal organization of parts within an experience, or by modeling external relations between instances of experience. A major next step for the structural approach is to integrate these two perspectives into an account of phenomenally unified global experience. In this paper, we describe these two types of structural models and how their category-theoretic formalizations contribute to the task of identifying the physical bases of phenomenal consciousness. We then propose a sheaf-theoretic framework that integrates these two approaches by mapping mereological parts of experience to empirical measures of their qualia. Through an application to the experience of visual space, we demonstrate that this framework enables a formal description of the structure of experience and conditions for phenomenal unity. We discuss how this integrative approach supports an empirical research program for investigating the relationship between local and global phenomenal qualities, and outline directions for future work toward a structural characterization of global phenomenal consciousness.

## 1. Introduction

Conscious experience comprises a “teeming multiplicity of objects and relations” (James, 1890) [1]: we experience a visual scene, sounds, as well as our thoughts, feelings, and bodily sensations simultaneously in a single moment. These experiences do not occur in isolation but as integrated parts of a single moment of consciousness, and their phenomenal qualities—what they feel like, or *qualia* (singular form *quale*) [2]—seem unified, such that their simultaneous experience gives rise to a distinct conjoint phenomenology of the total state of consciousness [3]. Such *phenomenal unity* is considered one of the most salient and defining features of consciousness, and one that any objective theory of consciousness must account for. In order to progress towards identifying the physical mechanisms that support unified states of consciousness, a precise description of its phenomenal character is necessary.

As with many problems in the study of consciousness, characterization of phenomenal unity remains a persistent challenge. Treating phenomenal unity as an additional phenomenal property falls into an infinite regress (see [4,5]), while explaining unity by appealing to the fact that experiential parts occur within a single encompassing state fails to be sufficiently informative about the conditions and mechanisms by which unity is achieved [5,6]. A more promising alternative is to treat phenomenal unity as a structural relation between experiential parts [5,7]. Then, phenomenal qualities of a unified state of experience can be defined as the properties that arise from this relational structure.

**Figure 1:**
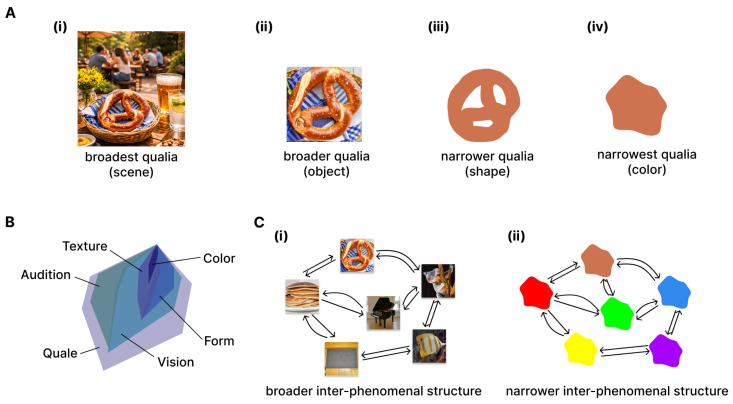
(**A**) The spectrum of narrow to broad qualia. Narrow qualia refers to the phenomenal qualities of a specific aspect of experience, while broad qualia refers to the phenomenal qualities of experience that includes those narrow qualia as its parts. We use the relational terms *narrower* and *broader qualia* to refer to the apparent hierarchy of phenomenal qualities. For example, the qualia of objects (e.g., colored shapes, **A**(**iii**)) may be considered narrower than the qualia of the object with those aspects as its features (**A**(**ii**)), but broader than the qualia of color (**A**(**iv**)). By this definition, qualia in the broadest sense correspond to the phenomenal qualities of the total field of consciousness (**A**(**i**)), while qualia in the narrowest sense correspond to experiences that cannot be further decomposed into narrower qualia that can be experienced separately, such as color (**A**(**iv**)). (**B**) A schematic diagram of *intra-phenomenal structure*, which represents the internal organization of a single experience. (**C**) Schematic diagrams of *inter-phenomenal structures*, which represent the structure of external relationships between instances of narrow and broad qualia. Depending on the objects of the structure, it can be a broader inter-phenomenal structure (e.g., of objects, **C**(**i**)), or a narrower inter-phenomenal structure (e.g., of color, **C**(**ii**)). (**B**) is reproduced with permission from [8].

This proposal necessitates specifying the precise mereological structure of phenomenal experience, i.e., the relations among local experiential parts and their relation to the global experience. More precisely, it requires characterizing the relationship between their phenomenal qualities: between the qualia of particular aspects of experience, such as color, odor, or emotions—referred to as *qualia in the narrow sense*, or *narrow qualia* [8,9]—and the qualia of their joint experience—referred to as *qualia in the broad sense*, or *broad qualia* [8,9] (Figure 1A). Both compositional and decompositional aspects of this relationship must be clarified [7]. That is, how do the narrow qualia of various phenomenal contents of experience combine to determine its broader quale, and how does the partitioning of broader quale, such as via selective attention, result in its constituent narrower qualia?

Generally, the relation between narrow and broad qualia exhibits a form of interdependence in that altering a local feature induces changes in the phenomenal quality of other local aspects, and in turn, that of the whole [7,10]. This is evident in many cases of feature integration (e.g., the influence of color saturation on perceived size [11]) and multisensory perception (e.g., the McGurk effect [12], sound-induced flash illusion [13]), as well as in sensory–conceptual interactions (e.g., sound-symbolism [14]) and various cases of context dependence (e.g., the effect of perceived illumination conditions on color, as seen in the checker-shadow illusion [15]). Such interdependence seems to come in degrees: in some cases, phenomenal qualities seem necessarily fused such that they cannot be experienced separately (e.g., as in the case of saturation and brightness of color [16] or pitch and loudness of sound [17]). In other cases, experiential parts seem more or less independent (e.g., the presence or absence of background music does not seem to affect the phenomenal quality of a visual object). Nevertheless, their conjoint phenomenology always possesses the phenomenal quality of unity—i.e., phenomenal unity is always “experientially manifest” [5]—such that the broad quale of the unified experience seems irreducible to a simple aggregate of the narrower qualia of its parts [1,6]. The relation between narrow and broad qualia must therefore accommodate varying degrees of such interdependence while accounting for their phenomenal unity. Clarifying this structural relation between narrow and broad qualia can help guide the identification of the mechanisms underlying *phenomenal binding*, which may provide one potential constraint for theories of consciousness [18].

Importantly, a structural characterization of phenomenal unity must distinguish phenomenal binding from *functional binding* [19,20,21,22]. Phenomenal binding concerns the integration of the phenomenal qualities into a unified conscious experience; in contrast, functional binding concerns the integration of perceptual information into coherent percepts, such as the binding of multiple properties as attributes of a unitary object (e.g., an object is perceived as both red-colored and circle-shaped) at the level of cognitive processing [21,23]. Functional binding of perceptual information does not by itself entail phenomenal binding, as it can occur without conscious experience [24] (e.g., [25]). Thus the mechanisms underlying functional binding at the level of cognitive processing may be related to, but not necessarily coincide with, the mechanisms that achieve the binding of phenomenal qualities into the unity of consciousness [19].

Because functional binding is often discussed in the context of feature binding (i.e., the integration of perceptual features such as color, shape, or orientation as attributes of a unitary object [26]), it is worth highlighting a further distinction between local and global levels of phenomenal unity. The features bound into a unitary percept of an object, when consciously experienced, are also phenomenally unified. That is, the narrower qualia of these features (e.g., redness of its color or circle-ness of its shape) are unified at the level of the object, as part of its broader quale. However, regardless of whether the phenomenal qualities of these features are experienced as parts of a unitary object, we still enjoy phenomenal unity at the level of the entire experience. Even if, for example, visual and auditory qualia are not experienced as attributes of the same object, they are nevertheless globally unified as part of a unified moment of consciousness and jointly determine its broadest quale [27]. The phenomenal binding of features into objects thus includes a form of local unity that is not necessary for global phenomenal unity. While conscious feature binding involves both functional binding and phenomenal binding [3,23], mechanisms underlying the local binding of features may not be sufficient to explain global phenomenal unity. This further highlights the distinction between the mechanisms of functional binding that support the integration of perceptual information and the mechanisms that support phenomenal unity. Given that contents of consciousness contain objects and events supported by various kinds of functional binding [26], it seems plausible that these processes partially share mechanisms with the binding of their phenomenal qualities. This has indeed motivated early work on the *neural correlates of consciousness* (NCC), which was informed by brain dynamics correlated with perceptual binding and integration (e.g., [28,29,30]). However, identifying the mechanisms that achieve the phenomenal unity of consciousness requires a careful distinction between phenomenal binding and other forms of binding that occur at different perceptual or cognitive levels.

The distinction between phenomenal and functional binding emphasizes the importance of describing phenomenal part–whole relations at the level of phenomenology [19], using phenomenological descriptions of the structure of experience and its phenomenal qualities. These descriptions must in turn be grounded in appropriate empirical measures of qualia for it to be a testable model of phenomenal unity. To this end, we turn to recent developments in *structural approaches to consciousness*, a methodological framework for better describing the phenomenal character of experience using mathematical structures [31].

Current structural approaches provide formal descriptions of qualia in two different ways: by modeling the internal organization of experiential parts within an experience (Figure 1B), or by modeling relative differences between instances of qualia (Figure 1C). We propose a framework for integrating these two types of qualia structures within a single coherent system, toward a model of the structure and phenomenal character of unified states of experience. We do so using category and sheaf theory, mathematical frameworks particularly well-suited for studying the composition of local structures into a coherent global whole.

In what follows, we first provide a brief overview of recent developments in the structural approaches in consciousness research (Section 2), and motivate the mathematization of phenomenological structures and the use of category theory as the appropriate formal language (Section Mathematizing Structural Descriptions of Qualia). We then describe the two types of structural descriptions and their mathematical formalizations as categories (Section 3). These two categories are then integrated into a unified framework, which offers a formal and empirical model of the phenomenal character of global experience (Section 4). We discuss how this proposed framework accommodates a range of possible relationships between measures of narrow and broad qualia and enables an empirical research program for investigating their structural organization (Section 5). We end with a discussion of limitations and directions for future research (Section 6).

## 2. Structural Approaches to Phenomenal Consciousness

The structural approach to phenomenal consciousness [31,32,33] is a research paradigm that utilizes mathematical structures to characterize the phenomenal aspects of experience, which are then proposed to constrain the identification of its underlying physical mechanisms. The goal of this approach is to overcome the limitations of the previously dominant correlational method for finding the NCC [34,35,36], which have relied on correlating some measure of conscious experience with co-occurring dynamics of the brain. Although this approach has led to many important insights, it has revealed at least two limitations in providing an explanatory account of qualia.

First, it falls short of characterizing the explananda of the problem of consciousness itself, which are the subjective, phenomenal aspects of experience. Most experimental methodologies in the NCC research program probe for mechanisms underlying the general presence of conscious phenomena, either in relation to conscious perception (e.g., detected vs. undetected stimuli) or conscious experience in general (e.g., wake vs. deep sleep, minimally conscious state vs. coma), based on behavioral measures of stimuli detection [31]. This leaves out the phenomenal character of experience, instead substituting it for its behavioral or functional correlates [37]. Although the neurophenomenological research program [38,39] directly incorporates phenomenological data, some methodological challenges remain. For example, neurophenomenological studies to date mainly focus on specific types or aspects of experiences (e.g., sense of self or agency in altered states of consciousness [40], interoception [41], or attention [42,43]) and are constrained by verbal reports that often require participant and interviewer training [44,45] or by measurement scales that restrict the characterization of experience to a selection of experiential dimensions (e.g., [46,47]). While both traditional NCC and neurophenomenology paradigms continue to play an essential role in bridging subjective and neural measures of consciousness, additional methodological approaches are needed to obtain a more comprehensive measure of qualia.

Second, the correlational approach cannot explain *why* a given pattern of neural activity corresponds to a particular subjective experience. Central to the notorious “hard problem of consciousness” [48] is the question of what non-arbitrary relationship explains the seemingly arbitrary co-occurrence of subjective experience and its physical correlates. That is, what is the systematic relationship that makes *this* particular neural activity co-occur with *this* particular subjective experience and not others? Traditional univariate approaches based on statistical correlations between neural dynamics and states of consciousness remain susceptible to spurious correlations [34]. A more principled account is necessary to move beyond a mere catalog of co-occurrences [36].

The current structural approach is suggested as a potential solution to these limitations. The core idea of this approach is to use mathematical structures to better describe phenomenal aspects of experience, and then to use these mathematical structures to guide the discovery of the systematic, non-arbitrary relationship between phenomenal and physical states [31,33,34,49,50]. Rather than associating an individual quale with a pattern of neural activity on a one-to-one basis, the structural research paradigm proposes to formalize the phenomenal–physical relationship at the level of their structures: *qualia structures*, which are structural descriptions of phenomenal experience, are related to structures of co-occurring states of their physical substrates (e.g., neural or informational), with a *structure-preserving map* [31,32,33]. Loosely speaking, a structure-preserving map between two structures is a map that preserves the relations among objects in one structure within the other (see Appendix A Definition A11). Thus, a structure-preserving map between the phenomenal and physical domains requires that the structure of physical substrates underlying phenomenal experiences preserve the relationship specified by the corresponding qualia structure. In this way, qualia structures impose constraints on the identification of their physical substrates [34,49], with structure-preserving maps capturing the principled relationship between co-occurring phenomenal and physical states. This hypothesized structure-preserving correspondence between phenomenal and physical structures builds on a longstanding lineage of philosophical thought (e.g., [24,51,52]). Contemporary structural approaches advance this hypothesis with an emphasis on the mathematical formalization of both domains and their relations, often employing more nuanced formal notions than those previously used. Ongoing work in this research paradigm therefore focuses on developing precise mathematical descriptions of qualia.

Currently, there are two ways in which phenomenal experience is characterized with mathematical structures [53]: one approach describes the internal organization of experiential parts within a single global state of experience (e.g., [54,55,56]) (Figure 1B), while another approach describes the external relations between instances of experience (e.g., [57,58,59,60]) (Figure 1C). We describe these two types of qualia structures in more detail in Section 3. Before turning to the structural descriptions themselves, we first present some motivations for their mathematical formalization and how this contributes to a principled physical account of qualia.

### Mathematizing Structural Descriptions of Qualia

Mathematical formalization offers several advantages for the structural research program. First, it provides a common bridging language with which to describe and compare across phenomenal and physical domains [36]. Second, the formal properties of mathematical structures, based on their set of relations, operations, or axioms, can be analyzed to derive further insights and predictions. Third, mathematical formalization allows these predictions to be generalized across domains that share the same structural form. A particularly useful mathematical framework that provides these advantages is that of *category theory*. Category theory [61,62,63,64,65] was developed for the study of structures and their relations, originally to clarify the structural parallels between seemingly disparate branches of mathematics. Given this, category theory is a promising choice as the formal language with which to describe the relationship between phenomenal and physical structures [36,66,67,68].

In category theory, a structure is represented as a *category*, which consists of a collection of *objects* and a collection of directed relations between them, called *morphisms* (also called *arrows* or *maps*) (Appendix A Definition A1). Objects can represent a wide array of entities, such as narrow or broad qualia, patterns of neural activity, or any mathematical structure such as sets, vertices, vector spaces, or topological spaces. Morphisms describe the relations between objects and can denote mathematical relations (e.g., functions, edges between vertices, structure-preserving maps) and qualitative relations (e.g., similarity, inclusion, or preference), as well as their quantitative measures (e.g., similarity ratings obtained from experimental procedures [69]) (for axioms that these relations must satisfy in order to constitute a category, see Appendix A Definition A1). This flexibility has led to many applications of categories to characterize the fundamental structure of various systems across a wide range of fields outside of mathematics (e.g., in quantum physics [70], cognitive science [71]). Within consciousness research, levels and contents of consciousness have been formalized as categories [72], and their relations to reports and measurements have been formalized as morphisms between categories [69,73].

Importantly, a category defines its objects in terms of their structural relations to other objects via their morphisms. This means that the domain-specific, internal details of the objects are abstracted away, leaving only a description of their structural relations. Thus, a category formalization of phenomenal experience will define its objects—such as narrow or broad qualia, or levels or contents of consciousness—in terms of their relations to other such objects without requiring detailed accounts of their intrinsic structure. Given these relations, new objects can be constructed so that they satisfy certain structural properties in relation to other objects (e.g., making a diagram *commute* (Appendix A Definition A2)). These new objects may correspond to essential structural features of the system. Applied to consciousness, representing the structure of experience as a category enables deriving fundamental features of consciousness, such as unity or composition, just by virtue of its mathematical structure without making assumptions about properties of experience [74]. Recent works that illustrate this approach are discussed in Section 3.1.

Because objects are defined in purely relational terms, these constructions can be generalized across domains, provided that the domains share the same structural characterization. Objects that occupy the same relational position across domains will satisfy the same structural properties, so a construction established in one structure automatically also applies in every domain with the same relational structure. This makes category-theoretic formalizations especially useful for clarifying shared structural properties across different systems. Thus, if phenomenal states and their underlying physical states are assumed to be *structurally equivalent* (Appendix A Definition A12) as required by the structure-preserving map, conclusions drawn in the phenomenal domain can be expected to hold in the physical domain. The same principle applies across phenomenal and stimulus domains, as well as between phenomenal states and reports, and so on. We provide an example to demonstrate this point in Section 4.1.5.

A growing body of work in consciousness science applies category theory to formalize the internal organization of phenomenal experience [56,75,76], external relationships between experiences [69,72,73], as well as neural or informational structures [67] and theories of consciousness [74,77]. Category structures and their mathematical principles have been used to explain compositionality and systematicity in cognition [71,78,79,80] and to formalize the axiomatic properties of consciousness asserted by Integrated Information Theory [74,81]. In the next section, we give a brief overview of existing applications of category theory to both internal and external structures of experience, before extending them to propose an integrative framework.

## 3. Two Types of Structural Descriptions of Qualia

### 3.1. Intra-Phenomenal Structures

The first type of structural description of qualia models the internal structural organization of an instance of phenomenal experience. These intra-relational qualia structures, which we term *intra-phenomenal structures*, aim to describe the mereological organization of experiential components within the total field of consciousness [82]. The mathematical formalization of intra-phenomenal structures can provide elegant accounts of invariant features of consciousness as consequences of its mereological structure, rather than as independently asserted assumptions. For example, recent work derives the phenomenology of the spatial extendedness of visual experience as a result of constructions in a category of regions of space related with inclusions [74]. Another study clarifies puzzling aspects of time consciousness, such as the simultaneously unchanging and transient nature of the present moment, through a category formalization [56]. Others have proposed formal models of experience as a category of qualitative states and processes, whose structural properties are used to derive phenomenal distinctions between contents of experience or the privacy of phenomenal experience [76].

Although the formal and empirical characterization of intra-phenomenal structures is in its early stages, the ultimate goal of this approach is to impose structural constraints on the identification of the NCC at the level of individual states, such that their physical bases also realize the part–whole relations specified by the intra-phenomenal structures [10]. This idea is reflected in the approach taken by Integrated Information Theory (IIT) [81], which first posits a phenomenal structure of experience (Figure 1B) that accounts for the fundamental properties of consciousness. This structure is composed of substructures corresponding to experiential components (“phenomenal distinctions”), along with relations that bind them into a unified whole. These phenomenal substructures and their relations are hypothesized to be mirrored in the physically instantiated structure that underlies this experience [54]. However, the intra-phenomenal structure alone may not be sufficient to impose such constraints. Specifying the internal organization of an experience may capture some structural aspects of its phenomenal character, but it does not provide an exhaustive description of *what it feels like* to experience the contents of the experience. For example, while the experience of an object may be described as a structured composition of its shape and color, this structural description does not specify what it feels like to see its color, shape, or their combination as an object. In this sense, intra-phenomenal structures might “specify the forms of experiences while leaving open how to color in those forms” with measures of their phenomenal qualities [83]. Furthermore, existing models of intra-phenomenal structure remain largely theoretical and lack direct connection to phenomenological data. For these models to also account for phenomenal quality in a way conducive to a falsifiable empirical analysis, they must be integrated with appropriate empirical measures of phenomenology. This motivates the second type of structural approach, which aims to provide measures of phenomenal qualities by specifying external relations between instances of qualia.

### 3.2. Inter-Phenomenal Structures

The second type of structural approach aims to describe the phenomenal quality of experience through its relations to that of other experiences. These relations are encapsulated in inter-relational structures of qualia, which we term *inter-phenomenal structures*. Instances of phenomenal experience are placed within a relational structure [53], and the relative position of each experience in this structure is taken as the measure of its phenomenal quality. For example, an inter-phenomenal structure of color experiences (often referred to as *color qualia spaces* or *color qualia structures*, Figure 1C(ii)) consists of a set of phenomenological relations, such as perceived similarity, between instances of color experiences. Then, the phenomenal quality of seeing the color red—“redness of red”—is defined in terms of its relations to the phenomenal qualities of other color qualia in this structure, e.g., as being “more similar to orangeness of orange than to blueness of blue”. Inter-phenomenal structures can be constructed for other types of experience to describe narrower or broader qualia, by specifying the external phenomenal relations in a similar manner. For example, an inter-phenomenal structure of object experiences (Figure 1C(i)) would characterize the broader qualia of objects through their relative positions within a broader, object qualia structure.

**Figure 2:**
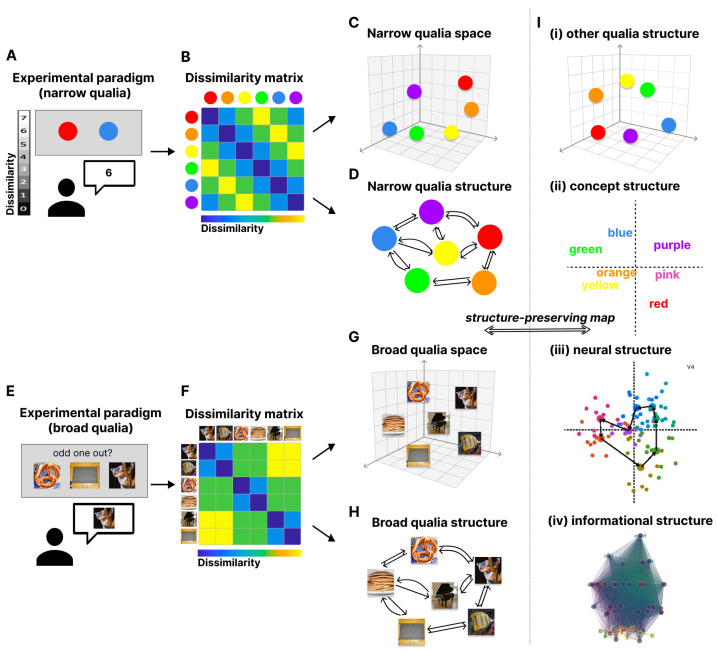
(**A**) Constructing inter-relational qualia structures and spaces begins with collecting relative proximity measures of the phenomenal experience of a given set of stimuli in an experimental paradigm. (**B**) Measures of experiential proximity, such as perceived similarity, are stored in a proximity matrix, to which additional embedding algorithms or mathematical formalization can be applied to further reveal and visualize an underlying relational structure. This relational structure can be embedded in a metric space, such as via multidimensional scaling (MDS) (**C**). Alternatively, it can be represented in a more general mathematical construct, such as a category (**D**) whose directed arrows accommodate non-metric relationships between qualia (e.g., violations of symmetry (red arrows) or triangle inequality (blue arrows)). (**A**–**D**) describe the standard empirical procedure for constructing inter-phenomenal structures for narrow qualia, such as of color, sound, or odor. These steps can be repeated using stimuli that evoke broader qualia, such as objects, scenes, or entire moments of experience (**E**–**H**), to obtain a broader inter-phenomenal space (**G**) or broader inter-phenomenal structure (**H**). Inter-phenomenal structures are then compared to structures of interest for structural similarity, such as (**I**(**i**)) other qualia structures (e.g., of participants with color blindness [57]) or other relevant structures, such as (**I**(**ii**)) structures based on conceptual similarity (e.g., of color concepts [89]), (**I**(**iii**)) neural structures (e.g., of activity patterns in V1 versus V4 [90]), (**I**(**iv**)) informational structures (e.g., cause–effect structure [81]), or similarity structures inferred from layers of artificial neural networks (e.g., [91]). (**A**) is adapted from [57]. (**E**–**H**) use sample stimuli from the THINGS dataset [92]. **I**(**iii**) is reproduced with permission from [90] (Copyright 2009 Society for Neuroscience). **I**(**iv**) is reproduced with permission from [81].

This approach has been particularly amenable to empirical investigation. Relational measures between instances of qualia can be collected experimentally, for example by asking participants to report perceived phenomenal similarities between pairs of stimuli, such as color patches (Figure 2A) (see Section 5 for a discussion of experimental paradigms for measuring phenomenal relations). These measures of phenomenal relations are then stored in a dissimilarity matrix (Figure 2B). This process can be repeated for a large number of subjects and across groups (e.g., [57]). From this, an underlying relational structure can be inferred, which can be represented in a geometric space (referred to as *qualia space*; Figure 2C) through an embedding function such as multidimensional scaling (MDS [84]), or more generally in an abstract mathematical construct, such as a category (referred to as *qualia structure*; Figure 2D).

This approach offers methodological advantages for measuring qualia given the difficulty of obtaining complete descriptions of phenomenal character through behavioral measures or verbal report [31,85]. Inter-phenomenal structures are defined solely on the basis of the external relations between instances of qualia, which can be specified independently of the internal properties of each quale. This is reminiscent of the way representational dissimilarity matrices (RDMs) of neural activity patterns capture functional similarities without a complete mechanistic account of the underlying system [49], regardless of whether those similarity relations arise from the overlap of tuning curves, spatial proximity of neurons, or shared neural circuit architectures (e.g., [86,87,88]). Similarly, the phenomenal relations between qualia may arise from shared visual features, associated concepts, evoked emotions, or a combination of such factors, which are abstracted by a relational measure of phenomenal difference (e.g., “is similar to”). The set of all such relations between a quale and all other qualia captures its rich and often ineffable character, without requiring full specifications of individual experiences. Furthermore, a measure of phenomenal difference is relatively simple to collect, allowing intersubjective comparisons with a larger pool of participants. This makes the inter-phenomenal approach particularly conducive to building an objective, empirical description of subjective experience.

When formalized as a category, an inter-phenomenal structure contains qualia as its objects (e.g., redness of red, orangeness of orange, etc.) and phenomenal relations as its morphisms, which may denote directed relationships such as “is nearly indistinguishable from” or “is less than or equally similar with respect to a reference” [66,72]. Directed relationships captured by the morphisms naturally accommodate potentially asymmetric relationships between qualia. This is a significant advantage over qualia spaces defined in metric spaces, whose axioms such as symmetry and triangle inequality are often violated by empirical measures of phenomenology (e.g., in similarity judgments of color [93,94,95], faces [59,96,97], and objects [98]).

Category formalizations of inter-phenomenal structures can then be related to other structures of interest, such as other qualia structures or relevant neural, informational, or artificial structures (Figure 2I) [57,58,66,91,99] by means of a structure-preserving map [31,32,33], such as a *functor* (Appendix A Definition A9). Recent applications of structure-based comparisons (e.g., Gromov–Wasserstein optimal transport [91]) between qualia structures (e.g., color qualia structures between adult and developmental groups [58] and between color-typical and color-blind participants [57]; between color qualia structure and the structure of stimuli features [91] or of semantic labels [89]) revealed systematic relationships previously undetected by correlational methods such as representational similarity analysis (RSA). For structures of corresponding physical states, the structure-preserving map requires that the relations specified in qualia structures be preserved. For example, the structure of neural activity patterns that support color qualia must be one in which the pattern of neural activity that instantiates the quale of red is more similar to that for orange than to that for blue [32,33,66,100]. The structure-preserving map thus becomes the basis of an account of the non-arbitrary link between co-occurring phenomenal and physical states [32,34,49]. This allows the differentiation between physical correlates that truly underlie phenomenal experience from those that are relevant for, say, stimulus processing [49,90].

Thus far, relational structures of perceived similarity, particularly those relevant to structural approaches to consciousness, have been specified for local features of experience, such as color [57,101,102], motion [103], sound [104,105], odor [106], faces [59], or emotions [107,108]. Although focus on narrow qualia is a reasonable starting point for establishing empirical and theoretical foundations, an inter-phenomenal structural description of broad qualia is necessary to progress towards identifying the NCC that supports the phenomenal character of not only features but states of conscious experience.

The most straightforward extension of the inter-phenomenal structural approach to broad qualia may be to apply the same empirical methodology (outlined in Figure 2A–D) to stimuli that evoke broader qualia, such as objects [109] or scenes [110,111] (Figure 2E–H). However, an inter-phenomenal structure does not, by definition, specify the relationship between the qualia of an experience and the qualia of its constituent features. For example, the inter-phenomenal structure of object qualia may provide a description of what it feels like to see an object, but does not explain how that experience is structured and how the broader quale of the object relates to the narrower qualia of its color and shape. That is, a broader inter-phenomenal structure alone leaves unspecified the relationship between broad and narrow qualia. A complete description of the phenomenal character of unified conscious experience therefore calls for an integration of inter- and intra-phenomenal structural approaches.

## 4. Mathematical Model of Broad Qualia

The distinction between intra- and inter-phenomenal structures makes evident that the two structural projects are not only compatible but significantly complementary [10]. From a category-theoretic perspective, these two types of structural models reflect a notion of *duality* (Appendix A, Definition A4) [74]: the “internal” characterization of experience given by intra-phenomenal structure mainly concerns objects and arrows within a category, while the “external” characterization of experience given by inter-phenomenal structure concerns a category’s directed relationship with other categories. Bridging these dual perspectives can help achieve a more complete structural description of the phenomenology of global states of consciousness.

**Figure 3:**
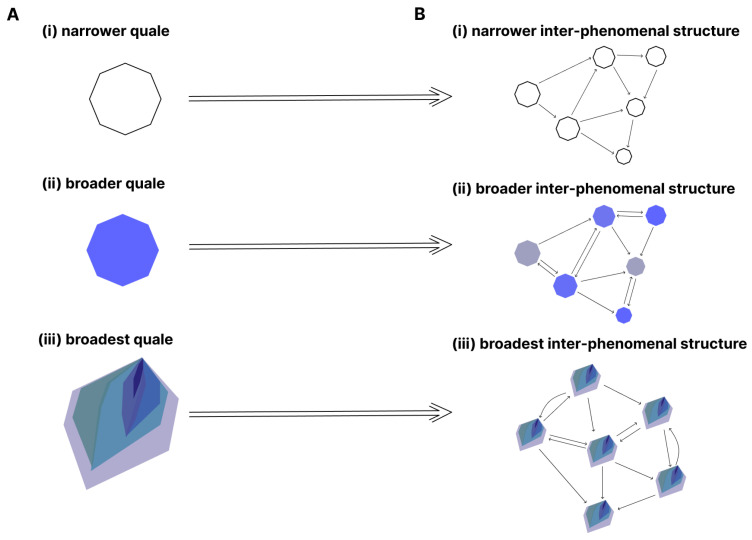
We hypothesize a model of the phenomenal character of global states of consciousness (i.e., broadest qualia) by integrating categories of inter- and intra-phenomenal qualia structures using a functor, called a presheaf. The presheaf (double-lined horizontal arrow) maps each mereological part of experience, from narrow to broad aspects (**A**), to measures of its phenomenal qualities characterized by the corresponding inter-phenomenal structure (**B**). For example, the phenomenal quality of a moment of experience, i.e., broadest quale (**A**(**iii**)) is described by its position within the inter-phenomenal structure that specifies its phenomenal relations to other moments of experiences (**B**(**iii**)). The narrower phenomenal quality of a colored shape that constitutes a part of this experience (**A**(**ii**)) is described by its position within the narrower inter-phenomenal structure that specifies relations among colored shapes (**B**(**ii**)). Similarly, the narrower phenomenal quality of its shape (**A**(**i**)) is described by its position within the narrower inter-phenomenal structure that specifies relations among shapes (**B**(**i**)). Arrows shown in the inter-phenomenal structures are schematic and do not correspond to measured relations. (**A**(**iii**)) is reproduced with permission from [8].

The most natural way to integrate intra- and inter-phenomenal structures is to map each mereological part of experience, given by the former, to measures of its phenomenal qualities, given by the latter. That is, given the internal organization defined by an intra-phenomenal structure, the phenomenal qualities of each experiential aspect can be specified by the inter-phenomenal structure of its contents. Through experimental procedures such as those described in Figure 2A,E, a hierarchy of inter-phenomenal structures can be defined for a single experience, from its narrowest (Figure 3A(i),B(i)) to broadest aspects (Figure 3A(iii),B(iii)). Thus, a full specification of the phenomenal character of a unified global experience must integrate these locally specified inter-phenomenal structures (Figure 3B(i–iii)) into a single coherent system. The organization of these structures is determined by the organization of experiential parts, specified by the intra-phenomenal structure (Figure 3A(i–iii)). Thus, modeling the phenomenal character of global experience entails formalizing the structural relations among inter-phenomenal structures across the hierarchy of its narrow to broad qualia.

This integrative model must account for the possible interactions between narrower qualia that result in a distinct broader quale. These interactions may manifest as conflicts between measures of phenomenal qualities specified at narrower versus broader levels of experience. For example, the phenomenal quality of the size of an object given the experience of its shape may differ from the phenomenal quality of its size given the experience of both its shape and its color [11]. These discrepancies may be reflected in data as differences in similarity judgments or misalignment of their underlying relational structures. Such conflict between narrow qualia that hold locally for particular aspects of experience and broad qualia that persist globally [112] must be reconciled in a model of global experience.

A mathematical framework for resolving tensions between locally and globally specified data is given by *sheaf theory* [113,114,115]. Generally speaking, sheaf theory provides a systematic framework for specifying the conditions under which locally specified data structures can be consistently combined—or fail to combine—into a global whole [115], where the etymology of the term *sheaf* (plural *sheaves*) reflects the idea of “naturally tying together” bundles of data. Sheaf theory thus seems appropriate as the mathematical framework with which to investigate the conditions for the phenomenal binding of experiential parts into a global experience. In what follows, we present how this framework can be used to formalize the compositional and decompositional relationship between measures of narrower and broader qualia. Specifically, we hypothesize that an empirical and formal study of the phenomenal character of global states of consciousness can be achieved by relating categories of inter- and intra-phenomenal qualia structures with a special type of functor called a *presheaf* (Appendix A Definition A13). We show that the presheaf maps each experiential part to the measures of its phenomenal qualities (Figure 3, horizontal arrows), and that this integrates empirical models of local and global phenomenal qualities into a testable model of global phenomenal experience. The following sections introduce formal notations for precision. Readers less familiar with the formalism may be interested in the brief summaries that begin with “Intuitively,” in which we restate the key ideas in more accessible terms.

### 4.1. Sheaf-Theoretic Framework for Broad Qualia

#### 4.1.1. Intra-Phenomenal Structure as a Topological Space as a Category

**Figure 4:**
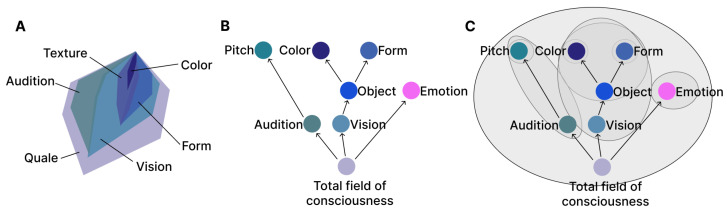
Intra-phenomenal structure as a preorder category and topological space. The inclusion relationship between aspects of experience (**A**) can be represented as a category, whose objects are broad to narrow aspects of experience (e.g., experience of vision, of object, of color, etc.) and morphisms are directed arrows indicating a subsumptive relationship (e.g., experience of vision subsumes experience of objects, which in turn subsumes experience of color) (**B**). (**C**) This preorder structure induces an *Alexandroff topology* that describes the relative proximity among experiential aspects. A topological space can be considered a category whose objects are open sets that correspond to experiential aspects and whose morphisms are inclusion relations between them. (**A**) is reproduced with permission from [8].

We start with a simple intra-phenomenal qualia structure that expresses the mereology of experience. We define a conscious experience E with experiential parts A,B,C,… as a category whose objects are aspects of experience, ranging from narrow (e.g., color experience) to broad (e.g., visual object experience), and whose morphisms indicate directed relations such as “AsubsumesB” or “BisexperiencedaspartofA” (Figure 4B). This captures the general subsumptive relation between phenomenal qualities of experience, that “experienceAsubsumesexperienceB” (B→A) when what it is like to have experience *B* is an aspect of what it is like to have experience *A* (Figure 4A) [3]. It is plausible that this subsumptive relationship is transitive: for example, the perceptual experience of a colored shape in space subsumes the experience of its color, which in turn subsumes the experience of its location. Therefore, the perceptual experience of a colored shape also subsumes the experience of its location. Generalizing this relationship, necessarily for all phenomenal aspects of experience A, B, and *C*, “if *A* subsumes *B* (B→A) and *B* subsumes *C* (C→B), then *A* subsumes *C* (C→A) ”. Thus, category E consists of a *preorder* (Appendix A Definition A5).

Given a finite preordered set of phenomenal aspects, we can induce an *Alexandroff topology* (also called *specialization order topology* (Appendix A Definition A7)). The Alexandroff topology τ induced on the preorder of phenomenal aspects describes the narrower aspect subsumed by a common broader aspect (e.g., experience of color and shape are subsumed by the experience of colored shapes) as more proximal to each other than those that are not (e.g., experience of sound). Using this topology, we can now define the mereological structure of experience as an *Alexandroff topological space* (E,τ) (Appendix A Definition A8) (Figure 4C). A topological space can itself be considered a category, whose objects are *open sets* (also referred to as *regions*), which correspond to subsets of the set of phenomenal aspects U⊆E, and whose morphisms are inclusion relations. Topological spaces provide a flexible formal framework for modeling phenomenal spaces, as they specify relative proximity relations between elements without requiring a metric distance [112].

Defining the structure of experience as a topology requires that it includes as its elements all intersections and unions of open sets. If the open sets of this topology correspond to sets of phenomenal aspects, their intersections would correspond to shared phenomenal aspects. For example, the phenomenal experience of location is a common experiential aspect shared between perceptual experiences of color and shape. The union of open sets would then correspond to the broader experience that subsumes its elements. For example, the union of the narrower experience of color and shape corresponds to the broader experience of colored shape. To illustrate, we formalize the experience of visual space as a topological space. We use this as a running example to develop the proposed sheaf-theoretic framework.

*Example. Experience of the Visual Field as a Topological Space*

**Figure 5:**
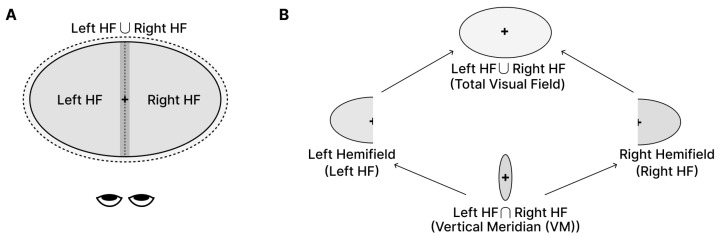
A simplified intra-phenomenal structure of the visual field. (**A**) Experience of the visual field can be subdivided into the left and right visual hemifields based on the point of fixation. The underlying topological structure is expressed in (**B**), which shows the inclusion relations where the experience of the total visual field (TotalVF) subsumes the experience of the left visual hemifield (LeftHF) and the right visual hemifield (RightHF). The intersection of the left and right hemifields corresponds to the vertical meridian (VM) through the point of fixation.

The phenomenal structure of visual space has previously been characterized as consisting of many overlapping regions and subregions [54]. The observation that the intersection and union of any two visual regions also constitute regions of the visual space [54] reflects the defining properties of a topological space (Appendix A Definition A6). Although the experience of the visual space likely comprises myriad such regions [116], we begin with a simplified construction that divides the visual field into two visual hemifields, i.e., left and right to the point of fixation (Figure 5A).

The experience of the total visual field subsumes the experience of the left and right visual hemifields, respectively: that is, LeftHF⊆TotalVF and RightHF⊆TotalVF. The intersection of the left and right visual fields corresponds to the experience around the vertical meridian (VM) through the point of fixation. The union of the left and right visual fields corresponds to the total, bilateral visual field. The topology of the mereological structure of the visual space can thus be formalized as τ={∅, {VM}, {LeftHF}, {RightHF}, {TotalVF}}, whose structure is expressed in Figure 5B.

Intuitively, the internal structural organization of experience can be formalized as a set of experiential aspects, where broader aspects subsume narrower ones, and shared experiential aspects define their overlap. For example, the experience of the visual field subsumes the experience of the left and right visual fields, respectively, which in turn each subsumes their intersection, which corresponds to the experience of the vertical meridian.

#### 4.1.2. Local Assignment of Phenomenal Qualities as a Presheaf

Given the topological space of experience, we now introduce a special kind of functor, called a *presheaf* (Appendix A, Definition A13), that attaches measures of qualia to each region of experience. A presheaf is simply a *contravariant functor* (Appendix A Definition A10) that maps each object and morphism in the domain category to those in the codomain category. In this framework, the domain category consists of the intra-phenomenal structure of an experience, and the codomain category consists of inter-phenomenal structures that characterize the phenomenal qualities of its contents. Thus, the presheaf maps each mereological part of experience to an inter-phenomenal structure that characterizes the qualia of its content, and maps the inclusion relations between experiential parts to relations between their corresponding inter-phenomenal structures.

**Figure 6:**
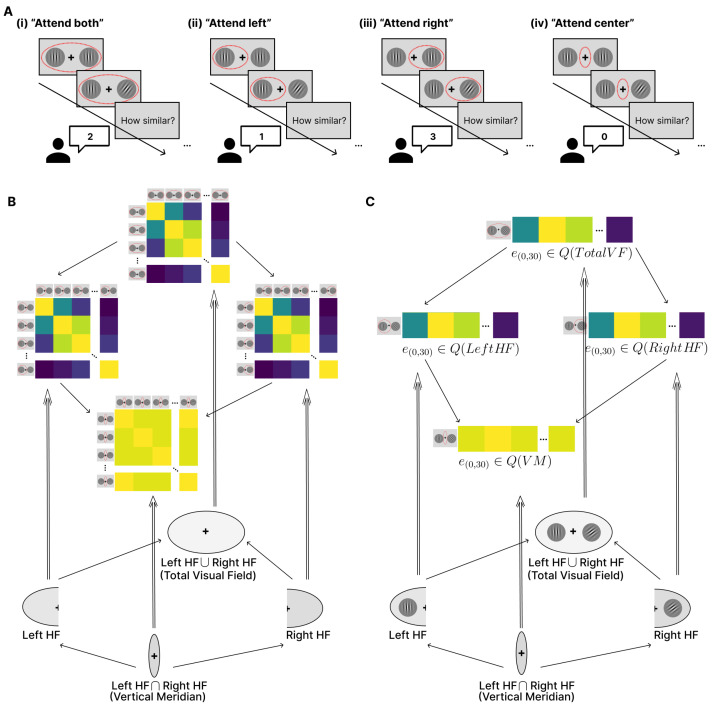
(**A**) Experimental procedures similar to that described in Figure 2 can be used to construct inter-phenomenal structures of broad (**A**(**i**)) and narrow qualia (**A**(**ii**–**iv**)) of experience. The broader inter-phenomenal structure is constructed from similarity judgments between trials involving simultaneous presentation of gratings across the entire visual field. Narrower inter-phenomenal structures are constructed from similarity judgments between trials while selectively attending to specific regions (e.g., left or right visual hemifields, or the vertical meridian; attended regions are shown in red). (**B**) The presheaf (vertical double-lined arrows) assigns, to each experiential aspect defined by the intra-phenomenal structure, the corresponding inter-phenomenal structure that specifies the measures of the phenomenal qualities associated with that region of the visual field. The objects in the codomain are dissimilarity matrices, related with restriction maps. (**C**) Each row of the dissimilarity matrix is a relational embedding that provides a measure of the phenomenal quality of experience in a single trial. These elements are called *local sections* and represent locally specified data assigned by the presheaf.

To illustrate, consider an experimental scenario in which the subject is presented with grating stimuli in each half of the visual field, with their orientations varying across trials (Figure 6A). The broader quale of the experience in the total visual field may be characterized by constructing an inter-phenomenal structure of similarity judgments between these trials, in which participants assess the experience of both gratings simultaneously (Figure 6A(i)). Narrower qualia in the left or right hemifields may be characterized by obtaining similarity judgments in a similar way, but while directing attention to either hemifield such that the judgments pertain specifically to the grating presented in the attended region (Figure 6A(ii,iii)). Similarly, the narrower qualia of the intersection of left and right hemifields can be characterized by the structure of similarity judgments of the experience along the vertical meridian (Figure 6A(iv)). Then, a presheaf, which we denote by *Q* (Figure 6B, double-lined arrows), maps each experiential region U⊆E to the corresponding inter-phenomenal qualia structure that describes its phenomenal quality (Figure 6B). The objects of this mapping, denoted Q(U), are mathematical objects that represent the inter-relational measures of qualia that are invariant under certain perceptually relevant transformations (e.g., of position, scale, illumination). Q(U) can take on different forms, such as dissimilarity matrices, their MDS-derived vector spaces, or category representations of the dissimilarity relations. In the present paper, we define them as empirically constructed dissimilarity matrices.

The elements of Q(U), called *local sections* (Appendix A Definition A14), represent the phenomenal quality of experience in a given trial. Local sections can be understood as measures of qualia taken in a particular “local” experimental or measurement setting. We define a local section $e_{\left(i,j\right)}^{U}\in Q\left(U\right)$ as the measure of qualia for the trial with stimulus pair $\left(\theta_{i},\theta_{j}\right)$, where $\theta_{i}$ and $\theta_{j}$ denote the angles of the left and right hemifield gratings, respectively, and the superscript *U* denotes the domain over which the local section is defined. Given that Q(U) is a dissimilarity matrix, $e_{\left(i,j\right)}^{U}$ corresponds to the relational embedding given by the row of Q(U) indexed by $\left(\theta_{i},\theta_{j}\right)$, which encodes pairwise dissimilarities between the experience of this trial and that of all other trials (Figure 6C).

To illustrate, consider an experience of a grating stimulus with a 0°-tilt relative to vertical in the left hemifield and a grating stimulus with a 30°-tilt relative to vertical in the right hemifield. The presheaf *Q* assigns to each experiential aspect a relational embedding that characterizes its narrow and broad qualia, from corresponding inter-phenomenal structures (Figure 6C):

- To {TotalVF}, the measure of broader quale obtained under simultaneous attention to the total, bilateral visual field $e_{\left(0,30\right)}^{TotalVF}\in Q\left(TotalVF\right)$;
- To {LeftHF}, the measure of narrower quale obtained under selective attention to the left hemifield $e_{\left(0,30\right)}^{LeftHF}\in Q\left(LeftHF\right)$;
- To {RightVF}, the measure of narrower quale obtained under selective attention to the right hemifield $e_{\left(0,30\right)}^{RightHF}\in Q\left(RightHF\right)$.

A presheaf can thus be regarded as a kind of “local data assignment” functor, as it assigns to each structural aspect of experience a measure of its phenomenal qualities. The resulting structure (Figure 6B) formalizes the hypothesized organization of experiential parts (domain, bottom of the figure) and the relationship between the data structures that represent their phenomenal qualities (codomain, top of the figure).

Intuitively, a presheaf assigns, to each aspect of experience as defined by the intra-phenomenal structure, a measure of the phenomenal qualities of that aspect. These measures of qualia are drawn from experimentally constructed inter-phenomenal structures.

#### 4.1.3. Decomposition of Broad to Narrow Qualia as Restriction Maps

In addition to attaching measures as objects, the functoriality of the presheaf maps the inclusion relations between experiential aspects to relations between measures of phenomenal qualities of those aspects, i.e., between measures of broader and narrower qualia. The contravariance of the presheaf reverses the directionality of the morphisms, such that the morphisms in its codomain map measures of qualia defined over broader aspects of experience to those defined over narrower aspects. If local sections are understood as representing the phenomenal properties that hold throughout an aspect of experience, then these morphisms can be understood as restriction maps that project these properties to the narrower aspects. For example, phenomenal qualities that hold over the entire visual field can be restricted to a part of the visual field, similarly to how values of a function can be restricted to a subset of its domain.

Given the subsumption of the left hemifield in the total visual field, i.e., LeftHF⊆TotalVF, measures of broad qualia specified over the total visual field are restricted to the left hemifield:

$$Q\left(TotalVF\right)\overset{\;\mathrm{restrict}\;}{\to }{Q\left(TotalVF\right)|}_{LeftHF}$$

Restriction of a particular measure of broad qualia defined over {TotalVF} to a narrower experiential aspect {LeftHF} results in a value in that subregion:

$$e_{\left(i,j\right)}^{TotalVF}\overset{\;\mathrm{restrict}\;}{\to }e_{\left(i,j\right)}^{TotalVF}{|}_{LeftHF}$$

Phenomenologically, this restriction operation corresponds to selectively attending to a narrower experiential aspect (e.g., the left or right hemifields) given a broader experience (e.g., of the total visual field). We present one possible formal definition of this restriction map, which then allows us to fully define the codomain of the presheaf as a category of dissimilarity matrices.

**Definition** **1** (Category DIS)**.** Let *X* be a set of elements, $X=\{x_{1},x_{2},\ldots ,x_{n}\}$.

Let $d_{X}$ be a function that assigns a dissimilarity value in the range [0,1] to each pair of elements $(x_{i},x_{j})\in X$, i.e., $d_{X}:X\times X\to \left[0,1\right]$.

We assume that this function is reflexive, i.e., $d(x_{i},x_{i})=0$ (an element has zero dissimilarity with itself), and symmetric, i.e., $d\left(x_{i},x_{j}\right)=d\left(x_{j},x_{i}\right)$ (pairwise dissimilarity is order-invariant). Although order effects have been observed in empirical studies of similarity judgments (e.g., [93,117]), we assume symmetry here for simplicity.

A category DIS of similarity matrices has as its objects $\left(X,d_{X}\right)$, whose morphisms $m:\left(X,d_{X}\right)\to \left(Y,d_{Y}\right)$ consist of a function m:X→Y such that

$$d_{X}\left(x_{i},x_{j}\right)\leq d_{Y}\left(m\left(x_{i}\right),m\left(x_{j}\right)\right)\;\mathrm{for}\;\mathrm{all}\;x_{i},x_{j}\in X$$

This means that the dissimilarity between a pair of elements $\left(x_{i},x_{j}\right)$ in matrix $\left(X,d_{X}\right)$ must uniquely map to a dissimilarity between the pair of elements $\left(m\left(x_{i}\right),m\left(x_{j}\right)\right)$ in matrix $\left(Y,d_{Y}\right)$, such that this dissimilarity between $\left(m\left(x_{i}\right),m\left(x_{j}\right)\right)$ cannot be smaller than the dissimilarity between $\left(x_{i},x_{j}\right)$.

Since the decompositional passage of phenomenological data flows from broad to narrow, this definition implies that the perceived dissimilarity of narrower qualia must be greater than or equal to the dissimilarity between the corresponding broad experiences. Although few experiments directly compare the magnitudes of perceived similarity at the level of whole stimuli and perceived similarity at the level of their features, this proposed definition is grounded in several lines of related empirical evidence.

First, it is relatively well established that attentional selection, induced by directing attention to (or away from) a spatial location or to a specific feature dimension of stimuli, not only affects sensory processing and therefore behavioral performance in stimuli detection and discrimination (e.g., [118,119,120]) but also the subjective appearance of stimuli. For example, spatial attention enhances basic visual features of stimuli in the attended location, such as its contrast or size [121,122,123]. Feature-based attention similarly induces perceptual distortions, whereby attended stimuli are perceived as more dissimilar from one another than unattended stimuli (e.g., for color [124] and orientation [125]). Furthermore, classical studies in multidimensional psychological scaling suggest that performance in similarity-based matching tasks is more precise when based on a single, separable feature dimension [126,127], and that selective attention to such dimensions modulates the structure of overall similarity and categorization [128,129].

Recent work suggests that such attention-induced changes in perceptual appearance may be explained by a global reshaping of the underlying geometry of the similarity structure of features [125,130] such that distances among attended features are expanded while those among unattended features are compressed. This idea is further corroborated by the recent demonstration that the withdrawal of attention weakens categorical separation of stimuli defined by dissimilarity judgments [59]. Together, these results suggest that attended stimuli may be experienced as more dissimilar than unattended stimuli. Based on these findings, we speculate a general relationship between overall and specific dissimilarity of experiences; that selective attention to a specific aspect of experience increases, or at least preserves, the perceived dissimilarity for that aspect that may have been less apparent when seen in their broader context.

Applying this definition to our example, the restriction map requires that the dissimilarity between broad qualia over the total visual field be less than or equal to the dissimilarity between narrower qualia over either visual hemifield. To formalize, let $\left(X,d_{X}\right)$ be the dissimilarity matrix that specifies the broad qualia of experiences over {TotalVF}. The set *X* consists of experiences associated with each trial, given a pair of grating stimuli. For example, $x_{\left(0,30\right)}\in X$ denotes the experience of a 0° grating in the left hemifield and a 30° grating in the right hemifield. Similarly, $x_{\left(30,30\right)}\in X$ denotes the experience of 30° gratings in both hemifields. The function $d_{X}$ specifies the perceived dissimilarity between these experiences, i.e., $d_{X}\left(x_{\left(0,30\right)},x_{\left(30,30\right)}\right)$.

Likewise, let $\left(Y,d_{Y}\right)$ be the dissimilarity matrix that specifies the narrower qualia of experiences over {LeftHF}, i.e., under attention directed to the left visual hemifield. The set *Y* consists of experiences associated with each trial, but under this attentional constraint. For example, $y_{\left(0,30\right)},y_{\left(30,30\right)}\in Y$ denote the experiences of the same pairs of grating stimuli while attending to the left hemifield. Again, the function $d_{Y}$ specifies the perceived dissimilarity between these experiences, i.e., $d_{Y}\left(y_{\left(0,30\right)},y_{\left(30,30\right)}\right)$. Then, the mapping between the dissimilarity matrices $\left(X,d_{X}\right)\to \left(Y,d_{Y}\right)$ requires that

$$d_{X}\left(x_{\left(0,30\right)},x_{\left(30,30\right)}\right)\leq d_{Y}\left(y_{\left(0,30\right)},y_{\left(30,30\right)}\right)),$$

which means that the dissimilarity between the narrower qualia under selective attention to the left hemifield is greater than or equal to the dissimilarity between the broader qualia of the corresponding full-field experiences. This restriction map defines a formal decompositional map from measures of broad qualia to those of narrower qualia.

Intuitively, the presheaf specifies the relations between measures of qualia across broader to narrower aspects of experience. We define this restriction map, based on empirical evidence, as a function that assigns dissimilarities between narrower qualia that are at least as large as those between the corresponding broader qualia.

#### 4.1.4. Phenomenal Binding as Gluing of Narrow Qualia

Defining this decompositional map enables the assessment of whether locally specified measures of phenomenal qualities are compatible with one another. In sheaf theory, data defined over regions of the topological space are considered compatible if their restrictions agree in a common subregion. In the case of our model of visual space, the common subregion between the left and right hemifields is that of the vertical meridian. Thus, locally specified measures of narrow qualia defined over left and right visual hemifields would be considered compatible if their restrictions agree in this region. Phenomenologically, restricting one’s attention to the vertical meridian from the left hemifield should result in the same narrower quale as restricting to the vertical meridian from the right hemifield.

**Figure 7:**
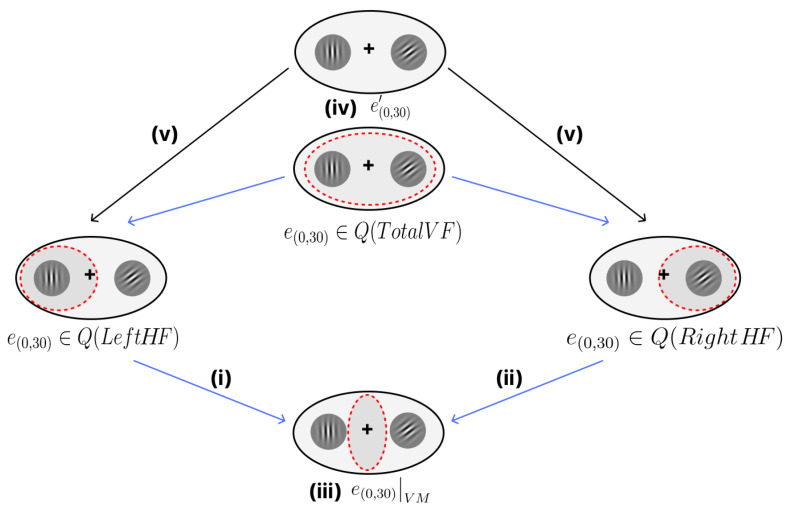
A sheaf diagram expresses the compositional and decompositional relations between measures of phenomenal qualities assigned to each experiential aspect. The local sections over the left and right hemifields can be evaluated for compatibility in their intersection at the vertical meridian (**i**–**iii**). If their restricted phenomenal qualities agree, these local sections can be glued into a global section over their union ($e_{\left(0,30\right)}^{\prime }$) (**iv**). This global section restricts to recover the local sections specified over the left and right hemifields (**v**). If these relationships hold for all regions of the topological space, the presheaf is said to satisfy the sheaf condition and thereby constitutes a sheaf. In this case, the global section ($e_{\left(0,30\right)}^{\prime }$) obtained by gluing compatible local sections coincides with the local section defined over the union ($e_{\left(0,30\right)}\in Q\left(TotalVF\right)$). A sheaf implies that the measure of broader quale over the total visual field is completely determined by, and decomposable into, measures of narrower qualia over the left and right hemifields. This is mathematically expressed by the commutativity of the diagram (blue arrows).

Mathematically, applying the restriction function defined above (Definition 1) provides the predicted measure of the narrower quale in the overlap. For example, consider again an experience of a trial given a 0° grating in the left hemifield and a 30° grating in the right hemifield. The restriction map $m_{VM,LeftHF}:Q\left(LeftHF\right)\to Q\left(VM\right)$ restricts the locally specified measure of narrow quale over the left hemifield $e_{\left(0,30\right)}^{LeftHF}\in Q\left(LeftHF\right)$ to the vertical meridian (Figure 7(i)):

$$e_{\left(0,30\right)}^{LeftHF}⟼e_{\left(0,30\right)}^{LeftHF}{|}_{VM}$$

Similarly, the restriction map $m_{RightHF,VM}:Q\left(RightHF\right)\to Q\left(VM\right)$ restricts the measure of narrow quale specified over the right hemifield $e_{\left(0,30\right)}^{RightHF}\in Q\left(RightHF\right)$ to the vertical meridian (Figure 7(ii)):

$$e_{\left(0,30\right)}^{RightHF}⟼e_{\left(0,30\right)}^{RightHF}{|}_{VM}$$

For visual experience that is unified across the visual field, these two restrictions are expected to result in identical measures of narrower quale at the vertical meridian (Figure 7(iii)), i.e.,

$$e_{\left(0,30\right)}^{LeftHF}{|}_{VM}=e_{\left(0,30\right)}^{RightHF}{|}_{VM}$$

Compatible local sections that agree in their overlap can be “glued together” to form a section over their union (*gluing of the sheaf condition*, Appendix A Definition A15). In this case, agreement at the vertical meridian allows the gluing of narrow qualia of the left and right hemifields into a measure of the broader quale over the total visual field (Figure 7(iv)):

$$\left(e_{\left(0,30\right)}^{LeftHF},e_{\left(0,30\right)}^{RightHF}\right)\overset{\;\mathrm{glue}\;}{\to }{e^{\prime }}_{\left(0,30\right)}^{TotalVF}$$

This section can be decomposed back to its subregions via the restriction map (Figure 7(v)) to infer measures of narrower qualia of the left and right visual fields:

$${e^{\prime }}_{\left(0,30\right)}^{TotalVF}\overset{\;\mathrm{restrict}\;}{\to }\left({e^{\prime }}_{\left(0,30\right)}^{TotalVF}{|}_{LeftHF},{e^{\prime }}_{\left(0,30\right)}^{TotalVF}{|}_{RightHF}\right)$$

which can then be assessed for whether they recover the independently specified local sections over those regions, i.e., whether

$$\left({e^{\prime }}_{\left(0,30\right)}^{TotalVF}{|}_{LeftHF},{e^{\prime }}_{\left(0,30\right)}^{TotalVF}{|}_{RightHF}\right)=\left(e_{\left(0,30\right)}^{LeftHF},e_{\left(0,30\right)}^{RightHF}\right)$$

If compatible local sections can be glued into a section over their union whose restriction recovers the original local sections, and if these conditions are satisfied throughout all regions of the topological space, the presheaf *Q* is said to satisfy the sheaf condition and therefore constitutes a *sheaf* (Appendix A Definition A15). This is mathematically expressed as the commutativity of the diagram in Figure 7 (blue arrows).

For a presheaf to be a sheaf means that the functor plays the role of a translation system that enables the systematic integration of local data structures into a global one. In the current example of the visual field, this means that compatible measures of narrower qualia of the left and right visual hemifields can be glued into a *global section* (Appendix A Definition A14) that represents the measure of a coherent broader quale of the total visual field. It further implies that the decomposition of this global section recovers the local sections over the left and right hemifields.

Intuitively, the restriction maps specified by the presheaf allow for the assessment of whether measures of narrower qualia, i.e., their inter-phenomenal structures assigned by the presheaf, are compatible as part of a single coherent system. Their compatibility is evaluated in shared experiential aspects defined by the intra-phenomenal structure. Because these measures are empirically derived, their compatibility can be assessed quantitatively. When local measures of narrower qualia are compatible, they can be glued together to infer the measure of broader quale, which can then be restricted back to test whether it recovers the original measures of narrower qualia from which it was inferred. If it does, the presheaf is said to satisfy the *sheaf condition* and thereby constitutes a *sheaf* (Appendix A Definition A15). The condition for compatibility in the overlap can be understood as a formalization of the condition for phenomenal binding of narrow qualia into a broader quale.

In the case of the visual field, empirical evidence supports this formalization. Given stimuli that are offset at the vertical meridian, participants exhibit aftereffects indicative of an adaptation mechanism that preserves alignment across the visual hemifields [131]. This suggests that compatibility at the vertical meridian may be necessary for a stable, unified percept of space across the two hemifields, a requirement formalized by the gluing condition of a sheaf.

#### 4.1.5. Hypothesized Relation Between Narrow and Broad Qualia as a Sheaf Diagram

Whether the functor *Q* between intra- and inter-phenomenal structures constitutes a sheaf is thus informative about the compositional–decompositional relationship that holds between narrow and broad qualia. Satisfaction of the sheaf condition means that the gluing of compatible local sections results in a global section of the broader quale, whose restrictions recover the originally assigned local sections. This implies that the measure of broader quale is wholly determined by, and decomposable into, measures of its narrower constituents. Phenomenologically, this suggests that the narrower qualia of aspects of experience fully determine the broader quale that subsumes them.

In contrast, failure of *Q* to satisfy the sheaf condition suggests that the narrower qualia arising from selective attention are insufficient to determine a coherent broader quale. Consider, for example, “impossible figures” such as the Penrose tribar [132], as discussed in [133]. At each junction of the tribar, the two adjoining segments are locally compatible in that they admit an interpretation of the figure that is consistent with the perceived configuration of the two segments. However, these pairwise compatibilities do not extend to a globally coherent configuration that is consistent with the perceived spatial configurations across all junctions. In this case, the functor *Q* that assigns measures of phenomenal qualities (here, the interpretation of the spatial configuration of the figure) forms a *presheaf but not a sheaf*: no global section—i.e., a globally consistent configuration—exists whose decomposition via restriction maps recovers all local sections—i.e., locally perceived configurations. This captures a form of contextuality [133,134], in which the figure’s global spatial configuration depends on the local observational context at each junction. Although impossible figures demonstrate contextuality at the level of cognitive interpretation, the same presheaf formalism can be applied to contextuality at the level of phenomenology. Phenomenologically, contextuality corresponds to a case in which a collection of narrow qualia fails to admit a broad quale that recovers them as simultaneous aspects of a single, coherent experience.

Binocular rivalry, a phenomenon often taken to reflect a failure of phenomenal unity, provides a compelling example of contextuality, a phenomenal “impossible figure”. In this case, the sheaf condition formalizes the condition for unity. In typical binocular vision, monocular percepts are fused into a coherent percept based on shared content in the central, binocular visual field. When monocular percepts are made to be sufficiently different, perception alternates between distinct global percepts, neither of which is jointly consistent with both monocular views. Formally, this can be expressed as a case in which no global section—i.e., a stable binocular view—exists that coherently integrates all local sections—i.e., monocular views—into a single unified experience.

**Figure 8:**
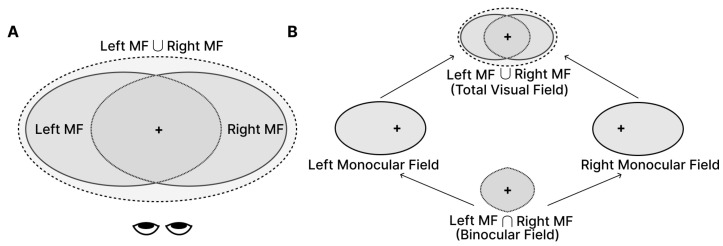
The structure of binocular vision can be formalized using the same sheaf diagram as the one used for visual space. (**A**) The structure of binocular vision can be subdivided into regions corresponding to the left and right monocular visual fields (denoted LeftMF and RightMF, respectively), whose union forms the total visual field. The intersection between left and right monocular visual fields consists of the central binocular visual field. The underlying topological structure is expressed in (**B**). Similarly to the fixation-based structure of the visual field, gluing of local sections over the left and right monocular fields depends on their compatibility in the overlap. Agreement of phenomenal qualities between left and right monocular vision in the shared binocular region gives rise to a unified visual experience over the total visual field. Binocular rivalry exemplifies a case in which incompatibility of two overlapping monocular percepts, induced by experimental manipulation, prevents their gluing into a stable visual experience.

Notice that in the case of binocular vision, the structure of visual space is specified in terms of the fields of view associated with each monocular view (Figure 8), rather than in terms of spatial regions defined by the locus of fixation as in the earlier examples (Figure 7). Although the two cases differ in the basis of their specification, both are formalized by the same sheaf diagram: with left and right subregions, together with their union and intersection (Figure 7 and Figure 8B). Given the same relational characterization, the domain-specific interpretations can be set aside, and the conclusions drawn from the fixation-based structure of the visual field can be generalized to this sensory-apparatus-based structure. In particular, compatibility of local sections in the intersection is likewise necessary to yield a unique global section. Agreement of phenomenal qualities in the overlap—here corresponding to the central, binocular visual field—supports the formation of a stable visual experience across the total visual field. Then, binocular rivalry can be diagnosed as a failure of gluing, in which incompatibility between the two overlapping monocular percepts prevents the existence of a stable, unique global section.

Intuitively, the sheaf diagram expresses a hypothesis about how the structure of experience (as specified by the intra-phenomenal structure) relates to measures of its phenomenal qualities (as specified by the corresponding inter-phenomenal structures). If this structure constitutes a sheaf, this indicates that the measures of narrower qualia assigned by the presheaf can be combined to fully determine the measure of the broader quale. On the other hand, if this structure does not constitute a sheaf, this suggests a form of contextuality, in which the measures of narrower qualia cannot be coherently combined into a unique measure of broader quale that is consistent with all measures of narrower qualia. When applied to cases such as binocular rivalry, violation of the sheaf condition may be diagnostic of phenomenal disunity.

The sheaf formalism can also be applied to information-theoretic measures of neurophysiological activity. On an algorithmic-information-theoretic reading, a sheaf structure that satisfies the gluing condition corresponds to a case in which a global state can be reconstructed from local components together with their compatibility relations, such that the structure admits a “compressive” description of the integrated organization of local measures. By contrast, a structure that fails the gluing condition, thereby yielding a presheaf but not a sheaf, corresponds to a case in which there exists no unique compressive description that organizes the local measures. The application of the sheaf formalism across these different levels of analysis, with their respective quantified measures, illustrates the value of category-theoretic formalization as a bridging language across domains. Because structures are defined relationally, the same formal definitions apply across domains, enabling the identification of potential structural similarities between phenomenal and physical states by means of a functor or other structure-preserving maps [66].

#### 4.1.6. Context Dependence as Violation of the Sheaf Condition

Finally, we consider the formalization of the possible interdependence between experiential parts. While the sheaf diagram developed above captures a hypothesized relationship between narrow and broad qualia, it does not explicitly characterize whether the broader quale reflects phenomenal qualities that arise from interactions among its constituent narrower qualia. As discussed in Section 1, the broader quale of experience is often shaped by complex interactions among experiential parts. This reflects the context dependence of measures of qualia, in that they are influenced by other, jointly experienced qualia within the broader experiential context [135]. Such effects often persist despite selective attention. For example, consider the Ebbinghaus illusion, in which the perceived size of an object is affected by the size of the surrounding objects (e.g., a shape surrounded by larger shapes appears smaller than when it is surrounded by smaller shapes). Attending to a particular target does not eliminate these contextual effects. Similarly, features of stimuli with so-called “integral dimensions” (e.g., saturation and brightness of color [16]) cannot be independently evaluated by attending to a single dimension, as judgments along one dimension remain sensitive to variations along the other. Recent empirical work further demonstrates that features of unattended stimuli affect the perception of attended stimuli (e.g., emotion of unattended faces influences the perception of attended faces [136]). Given these observations, a sheaf diagram whose local sections consist of measures of narrower qualia elicited by attending to a target in the presence of others, as in the examples above, may not by itself capture such context dependence. Instead, context dependence may be assessed using a sheaf diagram whose local sections consist of measures of narrow qualia elicited in isolation, in the absence of jointly experienced qualia.

**Figure 9:**
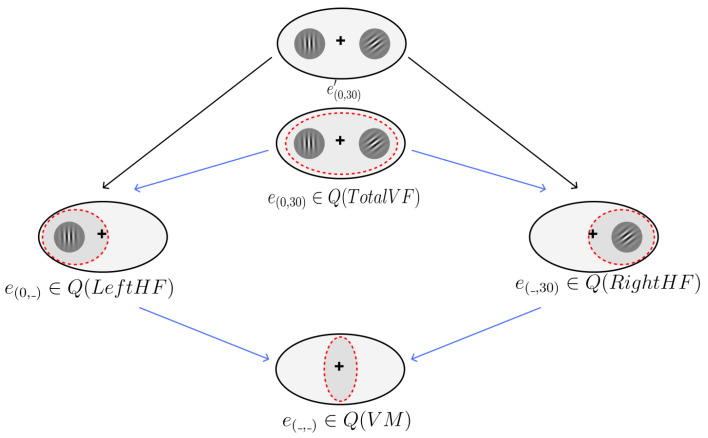
Whether narrower qualia interact to produce a broader quale with properties not present in its parts can be assessed using a sheaf diagram with measures of narrow qualia obtained under isolated viewing, rather than under selective attention in the presence of other experiential content. The “_” in the notations indicates the absence of a stimulus in the corresponding visual hemifield. Commutativity of this diagram (blue arrows) indicates that the broader quale can be inferred from the narrower qualia without effects of context dependence. By contrast, failure of commutativity provides evidence for context dependence or interactions between narrower qualia.

To assess whether the broader quale reflects context dependence, we construct a sheaf diagram whose local sections measure narrow qualia in the absence of other contents (Figure 9). In this case, narrow qualia are measured via selective attention given isolated presentation of a single grating stimulus. This contrasts with previously defined local sections, which measured narrow qualia given simultaneous presentation of both gratings (Figure 7). If there is minimal interaction between narrow qualia such that the presence of the grating in one visual hemifield does not affect the phenomenal quality of the grating in the other, this diagram should commute (Figure 9, blue arrows). That is, the broad quale of both gratings can be inferred from the gluing of these narrow qualia measured in isolation. If, on the other hand, there are significant interactions between narrower qualia, this diagram will fail to commute. This may be reflected in a discrepancy between the observed measure of broader quale specified over the union ($e_{\left(0,30\right)}^{TotalVF}$) and that inferred from the gluing of local sections over the left and right hemifields ($e_{\left(0,30\right)}^{\prime }$).

Commutativity of this diagram can be assessed empirically. For example, the observed dissimilarity matrix of broader qualia (containing $e_{\left(0,30\right)}^{TotalVF}$), obtained from simultaneous viewing of the stimuli, can be compared to the inferred dissimilarity matrix (containing $e_{\left(0,30\right)}^{\prime }$) derived from the narrower qualia measured under isolated viewing of the stimuli. Similarly, dissimilarity matrices of narrower qualia obtained from isolated viewing (containing $e_{\left(0,\_\right)}^{LeftVF}$ and $e_{\left(\_,30\right)}^{RightVF}$ in Figure 9, respectively, where “_” indicates the absence of a stimulus in the corresponding visual hemifield) can be compared to those obtained under selective attention during simultaneous viewing (the corresponding matrices in Figure 7). Agreement between these structures would suggest an absence of interaction between the narrow qualia of the two gratings. On the other hand, systematic discrepancies between these structures would indicate interactions between parts that are irreducible to a predictable combination of narrower qualia considered in isolation.

The possible difference in commutativity between the sheaf diagrams shown in Figure 7 and Figure 9 suggests that inferring the structure of broader qualia solely from measures of narrower qualia under conditions of isolated stimulus presentation may not accurately reflect the structure of experience. On the other hand, focusing only on attention-induced narrow qualia may obscure potential interactions between them. A complete account of the relationship between narrow and broad qualia must therefore consider measures of narrow qualia both in isolation and in the presence of other experiential content. Notably, as shown in Figure 7 and Figure 9, the same sheaf structure, instantiated with different local data structures, can be used to compare these different measures. This allows a systematic empirical investigation of contextual effects on measures of qualia, including those arising from experimental task demands [135,137]. We discuss this and its implications for future empirical research in Section 5.3.

Intuitively, to assess potential context dependence or interactions among narrower qualia, we construct a sheaf diagram whose local sections are specified using measures of narrow qualia of isolated phenomenal content, as opposed to those elicited under selective attention in the presence of other content. Failure of this diagram to form a sheaf provides evidence for contextual effects, whereby the broader quale reflects interaction-dependent phenomenal qualities that cannot be decomposed into the narrower qualia of its parts as measured in isolation.

## 5. Discussion

In this paper, we proposed a formal mathematical framework for modeling the phenomenal character of unified, global conscious experience. This is achieved by integrating two distinct yet complementary structural descriptions of phenomenal experience—of its intra-phenomenal part–whole relations and of its inter-phenomenal relations to other experiences—via a presheaf. The presheaf organizes inter-phenomenal structures, which provide empirical measures of qualia, according to the structure of experience specified by the intra-phenomenal structure. By application to the experience of the visual field, we showed that this framework can capture the compositional and decompositional relationships between narrow and broad qualia. We discuss the advantages and limitations of the current framework before concluding.

### 5.1. Hypothesis Testing with Sheaf-Theoretic Models of Qualia

The integration of intra- and inter-phenomenal structures enables an empirical research program for modeling phenomenal consciousness, extending previous approaches that treated these structures independently. For example, the intra-phenomenal structure of visual space has been characterized in terms of spatial regions related by inclusion, connection, and fusion [54], which was then used to hypothesize its underlying physical substrate. While this was a significant step towards a phenomenologically grounded identification of the NCC, these structural descriptions do not yet account for the phenomenal qualities of experiential content. As the authors of the study note, it is difficult to imagine experiencing visual space without the experience of its qualities [54]. Accordingly, a comprehensive description of phenomenal experience must capture not only its structural organization but also its phenomenal qualities. Incorporating inter-phenomenal structures via a presheaf provides such a description, by supplying empirical characterizations of phenomenal qualities that intra-relational accounts leave unspecified.

Grounding the description of phenomenal experience in empirical data supports a quantitative hypothesis testing of the proposed phenomenal structure. The sheaf diagram expresses a formal hypothesis about the organization of experience and its relation to empirical measures of qualia. This hypothesis can be evaluated by assessing how well observed measures conform to this structure [138]. Test statistics such as the *consistency radius* [114] can be used to quantify the degree of incompatibility among local sections in overlapping regions. A consistency radius close to zero indicates compatibility, in which case we fail to reject the hypothesis that the sheaf structure formalizes the structure of experience and its measurements. In contrast, a large consistency radius reflects substantial inconsistencies among locally specified data, providing quantitative evidence against the model. This would suggest that the observed data are unlikely to have been generated by a coherent sheaf structure.

This is a significant step towards a falsifiable scientific program for investigating the structure of experience, beyond models based solely on theoretical principles. In particular, this framework can be used to generate testable predictions for cases of phenomenal disunity. If the hypothesized structure of experience is on the right track, disunity observed in binocular rivalry or in split-brain patients may be diagnosed as violations of the sheaf condition—that is, cases in which a coherent global quale cannot be assembled from local ones. The precise formalization and empirical validation of these cases are left for future research.

### 5.2. Formalization of Phenomenal Unity

An empirically grounded model of global consciousness constructed with a presheaf offers key advantages as an account of phenomenal unity. First, phenomenal unity is not introduced as an additional phenomenal property that requires independent justification, but as a consequence of the phenomenal structure. Conditions for phenomenal binding are formalized as the compatibility condition for the gluing of local sections, determined by the topology of the intra-phenomenal structure. Whether this condition is satisfied is empirically tractable given the inter-phenomenal measures of qualia. Second, formalizing broad qualia in this way accommodates a spectrum of relationships between local and global phenomenal qualities. At one end of the spectrum, measures of broad qualia may be completely decomposable in the sense that they are fully determined by measures of narrow qualia. Such cases are captured by when the presheaf constitutes a sheaf. In many other cases, however, measures of broad quale may not be predictable from those of its narrow qualia. The current framework allows for the formalization of a range of possible relationships between narrow and broad qualia, without presupposing that phenomenal experience is either fully decomposable or entirely unanalyzable.

### 5.3. Limitations and Future Directions

The present framework is subject to several limitations, but provides a foundation for further development in future work.

#### 5.3.1. Partitioning of Experience

Most immediately, the application developed here is restricted to a highly simplified partitioning of the visual field into two regions, whereas the structure of visual experience constitutes a far richer organization (e.g., [116]), which may require an extension of the current mathematical formalization to general topological spaces on infinite sets. More broadly, the current framework is limited to a single sensory modality of vision, as well as to synchronic phenomenal unity within a moment of experience. Although the experience of visual space serves as a foundational example, its intra-phenomenal structure and the relations among their phenomenological data may not apply in other sensory modalities. The current framework must be extended to account for the phenomenal structure and qualities of multisensory experience and across the temporal dimension. For example, the intra-phenomenal structure of multisensory experiences may be specified according to organizational principles other than spatial location, such as temporal simultaneity or shared phenomenal content.

What organizational principles best specify the structure of experience is an open question, and one that the sheaf diagram expresses as a formal hypothesis. This points to promising future directions complementary to existing methodological approaches. For example, experiential features revealed from neurophenomenological analyses of subjective experience (e.g., [139]) or dimensions revealed from the analysis of large-scale similarity data (e.g., [109]) can be used to inform the formalization of intra-phenomenal structure. This yields a description of the internal structural organization of experience that goes beyond the characterization of qualia as an unstructured set of dimensions, such as a point in a multidimensional metric space [135,140].

#### 5.3.2. Experimental Procedures for Measuring Qualia

Another important limitation and direction for future empirical work concerns concrete experimental procedures for eliciting, measuring, and comparing narrow and broad qualia. The examples presented in this paper provide only a preliminary sketch and require further development and empirical validation. In particular, experimental methodologies for how to measure narrower qualia, how to measure broad qualia, and how to probe the relations between them, must be established.

Although the majority of current empirical work on inter-phenomenal qualia structures favor pairwise similarity ratings (e.g., [57,58,59]), it remains to be established whether this is the best-suited method for eliciting and obtaining relational measures of phenomenal qualities. Similarity, for instance, can be measured in various ways: directly as ratings on an ordinal scale for pairs of stimuli presented simultaneously [57] or sequentially [93], or indirectly inferred from the proportion of same-or-different judgments [141], odd-one-out judgments [109], spatial arrangement of multiple stimuli [142], or a list of attributes [143] (see also [144]). Some of these approaches have been shown to result in different relational structures (e.g., pairwise similarity versus spatial arrangement [145]), suggesting that different experimental methods may elicit judgments based on distinct underlying comparison criteria. For example, similarity measures based on prolonged viewing of stimuli (e.g., [109,142]) may be more reflective of similarity in terms of the conceptual characterization of the stimuli than their perceptual experience, although the nature of their relationship is debated [32,146,147,148,149]. Given this, identifying the most appropriate measures of phenomenal relations and the experimental paradigms for eliciting them is a particularly important topic for future empirical research.

More generally, the extent to which different experimental procedures affect measures of qualia remains to be empirically investigated. Previous findings suggest that variations in task demands, instructions, or methods of stimulus presentation may alter the measured structure of qualia. For example, experimental choices such as simultaneous versus sequential presentation of stimuli, as well as the presentation order, position, and format, have been shown to affect similarity judgments [93,94,150,151,152,153,154,155]. Furthermore, a recent study revealed different geometries underlying the structures of perceived similarity of musical pitch when tones were judged in isolation or within a melody [105]. These observations suggest that measures of qualia may also reflect context dependence arising from aspects of the experimental setting in which they are measured, such as the cognitive processes evoked by task demands or the presence of jointly presented stimuli [135]. The effects of specific experimental factors on measures of qualia should be empirically addressed in future research. The present framework may contribute to the systematic investigation of these effects, complementing existing approaches (e.g., [137,156]), for example through a formal comparison similar to one proposed between qualia structures obtained under different experimental conditions in Figure 7 and Figure 9.

Finally, a standard method for obtaining inter-relational measures of complex, broad experiences, as well as for comparing them to their component narrower qualia, has yet to be established. Most empirical research to date has focused on characterizing narrower qualia due to their relative empirical tractability; yet a characterization of phenomenally unified experience calls for empirical measures of broad qualia. However, given the difficulty of measuring the phenomenal qualities of broader experiences, few empirical studies construct inter-relational structures of broad qualia or compare these structures to those of narrower qualia under selective attention. Developing appropriate experimental methods for this task is thus a major outstanding challenge. Additionally, the decompositional relationship between broad and narrow qualia must be empirically investigated, along with an assessment of whether attentional paradigms are best suited for probing this relationship. We leave this for future work.

## 6. Concluding Remarks

A rigorous mathematical description of globally unified conscious experience is a prerequisite for bridging the gap between phenomenal and physical. This necessitates a formal description of the relations between the narrower qualia of experiential aspects, as well as their relations to the broad quale of the total field of consciousness. Without this, structural approaches cannot provide a complete physical account of consciousness that explains its fundamental properties, including phenomenal unity. The sheaf-theoretic framework proposed here is a step towards building a mathematically precise and empirically grounded description of global phenomenal consciousness. The mapping of intra-phenomenal structure of experience to inter-phenomenal structures that empirically characterize its phenomenal qualities generates testable predictions about the structure of experience and the conditions for phenomenal unity. Further development of such formalisms, based on mathematical principles and informed by the growing empirical literature on measures of qualia, will bring the field closer to a mature science of why consciousness feels the way it does.

## Appendix A

**Definition** **A1** (Category)**.** A *category* C consists of a collection of *objects* O(C)={A,B,…}, a collection of *morphisms* (also called *arrows* or *maps*) between objects M(C)={f,g,…} including *identity morphisms*, and a *composition* operation ∘.

A *morphism* between a pair of objects A,B∈O(C) is written as f:A→B, where *A* is the *domain* of *f* and *B* is the *codomain* of *f*. For each object in C, there is a self-referential *identity* morphism that maps an object to itself, i.e., $id_{A}:A\to A$. Given a pair of morphisms f:A→B and g:B→C whose codomain and domain coincide, the *composition* operation returns a *composite* morphism g∘f:A→C.
For C to be a category, its objects and morphisms must satisfy the *identity* and *associativity* laws:

- *Identity*: Composing any morphism with an identity morphism returns the morphism. That is, for every A,B∈O(C) and every morphism f:A→B, we have $f\circ id_{A}=f$ and $id_{B}\circ f=f$.
- *Associativity*: Composition is associative; that is, given three morphisms f:A→B, g:B→C, h:C→D, we have h∘(g∘f)=(h∘g)∘f.

Any collection of objects and morphisms can be made into a category so long as they conform to the identity and associativity laws. Objects may be vertices, sets, vector spaces, topological spaces, etc.; morphisms may be edges between vertices, functions, homomorphisms, structure-preserving maps, etc.

**Definition** **A2** (Commutative diagram)**.** Given f:A→B, g:B→C, and h:A→C, the composite g∘f need not be the same as *h*, but if it is, then the diagram is said to *commute*.

**Definition** **A3** (Opposite category)**.** $C^{op}$ denotes the *opposite category* of C, which is a category with the same objects as C but with morphisms pointing in the opposite direction. That is, a morphism f:x→y in $C^{op}$ is the same as f:y→x in C.

**Definition** **A4** (Duality)**.** There are two related notions of duality in category theory.

1. *Duality by arrow reversal.* Given a category C and its opposite category $C^{\mathrm{op}}$, a construction in C has a corresponding *dual construction* in $C^{\mathrm{op}}$. Classical examples include limits and colimits, products and coproducts, and terminal and initial objects. Dual constructions are the same construction viewed in the opposite category.
2. *Directional or oppositional duality.* More broadly, two constructions may be described as *dual* when they are opposed in direction. For example, a pair of functors F:C→D and G:D→C may be considered dual in the sense that they relate the same categories in opposite directions. This notion of duality reflects a contrast in the role or orientation of mappings, and is weaker than the notion of duality by arrow reversal [74].

**Definition** **A5** (Preorder)**.** A preorder (X,≤) consists of a set *X* and a binary relation ≤ that satisfies the following conditions:

- Reflexivity: For every x∈X, x≤x;
- Transitive: If x, y, z∈X with x≤y and y≤z, then x≤z;
- There is at most one arrow between two objects.

**Definition** **A6** (Topology and topological space)**.** A *topology* on an *underlying set X* is a collection T, whose subsets U⊆X are called *open sets* of T. An *open set* is a generalization of an open interval and provides a notion of nearness. A *region* of the topological space corresponds to an open set. For T to be a topology, its open sets must satisfy the following conditions:

- The empty set ∅ is an open set and is an element of T.
- The entire set *X* is an open set and is an element of T.
- The union of any number of open sets are open sets and is an element of T (closed under arbitrary unions).
- The intersection of any finite number of open sets are open sets and is an element of T (closed under finite intersections).

A *topological space* is the pair (X,T), i.e., a set *X* equipped with open sets. A topological space can itself be considered a category, with open sets as its objects and inclusions as its morphisms.

**Definition** **A7** (Alexandroff topology)**.** Let (X,≤) be a finite preordered set. The *Alexandroff* (or *specialization order*) *topology* on *X* is the topology whose open sets are upwards-closed sets of the preorder, that is,

τ=U⊆X|∀x∈U, x≤y

For example, consider the perceptual experience of a colored shape, whose subsumptive relationship between its phenomenal aspects can be described as a preorder:

$$E_{Location}\leq E_{Color}\leq E_{Shape}\leq E_{ColorShape}$$

This induces a specialization order topology

$$T_{ColoredShape}=\left\{\emptyset ,\left\{Location\right\},\left\{Color,Location\right\},\left\{Shape,Location\right\},\left\{Shape,Color,Location\right\}\right\}$$

which indicates that the phenomenal experience of color is “closer” to the experience of location than to that of shape, and “closer” to the experience of shape than to that of emotion (an experiential aspect outside this colored-shape hierarchy). In this way, the topological space of phenomenal experience describes the intra-phenomenal structural organization of its narrow to broad phenomenal qualities.

**Definition** **A8** (Alexandroff topological space)**.** An Alexandroff space is a finite topological space whose underlying set is a finite set. A topological space (X,τ) is called an Alexandroff topological space if the arbitrary (as opposed to finite) intersections of open sets are open.

**Definition** **A9** (Functor)**.** A *functor* is a map between two categories that translates objects and morphisms in one category to those in the other category while preserving structural relations between the objects. Formally, given two categories C and $C^{\prime }$, a functor $F:C\to C^{\prime }$ maps each object and morphism in C to $C^{\prime }$, that is, each object *A* in C is mapped to F(A) in $C^{\prime }$, and each morphism f:A→B is mapped to F(f):F(A)→F(B) in $C^{\prime }$. The functor must preserve identities and compositions:

- *Identity*: For any object A∈O(C), $F\left(id_{C}\right)=id_{F(C)}$;
- *Compositionality*: For every pair of compatible morphisms f,g∈M(C) where f:A→B and g:B→C, we have F(g∘f)=F(g)∘F(f).

**Definition** **A10** (Contravariant functor)**.** A contravariant functor reverses the direction of arrows in the codomain. Thus a contravariant functor F:C→D can be understood as a functor from the opposite category $C^{op}\to D$.

**Definition** **A11** (Structure-preserving map)**.** Loosely speaking, a structure-preserving map between two structures is a map that preserves the relations among objects in one structure within the other. Strictly speaking, a structure-preserving map may take different formal meanings in different mathematical contexts; for example, it may refer to a *functor* (Appendix A, Definition A9) in category theory, a homomorphism in algebra, or a continuous function in topology; in each case, the map preserves some operations and relations across structures. In the main text, we use the term in a general sense without committing to a particular formalization.

**Definition** **A12** (Structural equivalence)**.** Loosely speaking, *structural equivalence* refers to the preservation of the relevant relational structure across domains. In a strict category-theoretic context, different formal notions capture varying degrees of structural similarity, ranging from strong notions such as identity and categorical isomorphism, to weaker notions such as categorical equivalence, the existence of an adjunction, and the existence of a functor [66]. In the main text, we use the term in a broad sense without committing to a specific formal notion of equivalence.

**Definition** **A13** (Presheaf)**.** Generally, a *presheaf* on a topological space (X,T) is a contravariant functor $F:T^{op}\to \mathrm{Set}$, which maps to each open set U∈T a set F(U), called *sections* over *U*, and to each inclusion V⊆U a *restriction morphism* $\rho_{V}^{U}:F\left(U\right)\to F\left(V\right)$ that satisfies the identity and compositionality laws of a functor. That is,

- *Identity*: For each open set *U*, the restriction morphism $\rho_{U}^{U}:F\left(U\right)\to F\left(U\right)$ is the identity morphism $id_{F(U)}$.
- *Compositionality*: For open sets W⊆V⊆U, restriction commutes with composition of morphisms, i.e., $\rho_{W}^{V}\circ \rho_{V}^{U}=\rho_{W}^{U}$.

**Definition** **A14** (Local and global sections)**.** Let F be a presheaf on a topological space *X*. For any open set U⊆X, an element s∈F(U) is called a *local section* of F over *U*. These represent value assignments specified on the subregion *U*. Typically, a *global section* of F refers to a section over the entire space, i.e., an element s∈F(X). For an arbitrary presheaf, the set of local sections over the union of open sets may differ from the set of global sections that results from the gluing. A presheaf F is a *sheaf* if compatible local sections uniquely determine a global section. For a sheaf, the set of local sections defined over the union is the set of global sections. In this paper, we reserve the term global section to only refer to the coherent global data that results from a sheaf.

**Definition** **A15** (Sheaf)**.** Given a topological space (X,T) with ${\left\{V_{i}\right\}}_{i\in I}$ as the *open cover* of *U*, and a presheaf on that topological space $F:T^{op}\to \mathrm{Set}$, consider a sequence $s_{1},\ldots ,s_{n}$ where each $s_{i}\in F\left(V_{i}\right)$ is a section over $V_{i}$. F is a *sheaf* if it satisfies the following *sheaf condition* for every cover:

- *Gluing* (existence): If for all i,j there is a (local) section $s_{i}\in F\left(V_{i}\right)$ and a section $s_{j}\in F\left(V_{j}\right)$ that agree throughout their intersection, i.e., $s_{i}{{|}_{V_{1}\cap V_{2}}=s_{j}|}_{V_{1}\cap V_{2}}$, then there exists a global section s∈F(U) that agrees with the local section when restricted to the subregions, i.e., ${s|}_{V_{i}}=s_{i}$.
- *Locality* (uniqueness): The global section is unique, i.e., if there are sections s,t∈F(U) where ${s|}_{U_{i}}{=t|}_{U_{i}}$, then s=t.

That is, to regions {V,W}⊆U and {V∩W}≠∅, the presheaf will assign local sections to each region, i.e., $\left\{s_{1},\ldots ,s_{n}\right\}\in F\left(V\right)$ and $\left\{t_{1},\ldots ,t_{n}\right\}\in F\left(W\right)$. These local sections can be restricted to their intersection, i.e., a section $s_{i}\in F\left(V\right)$ can be restricted to $s_{i}{|}_{\left(V\cap W\right)}$; similarly, a section $t_{i}\in F\left(W\right)$ can be restricted to $t_{j}{|}_{\left(V\cap W\right)}$. Some of these sections might match in the intersection, i.e., for some $i,j,s_{i}{{|}_{\left(V\cap W\right)}=t_{j}|}_{\left(V\cap W\right)}$. These are called a matching family of sections. Given such matching families of local sections, the presheaf *F* is a *sheaf* if there exists a unique section u∈F(V∪W) whose restriction to the intersection recovers the originally assigned local section, i.e., ${u|}_{V}=s_{i}$ and ${u|}_{W}=t_{j}$. Note that for finite topological spaces such as the one considered in the present paper, the locality condition automatically holds when the gluing condition holds [114]. For arbitrary topological spaces on an infinite set, the two conditions must be established separately for the presheaf to be a sheaf.
